# Reply to: The impact of thermodynamics when using a catalyst for conventional carbon capture solvent regeneration

**DOI:** 10.1038/s41467-023-39695-9

**Published:** 2023-07-13

**Authors:** Masood S. Alivand, Geoffrey W. Stevens, Kathryn A. Mumford

**Affiliations:** 1 Department of Chemical Engineering, The University of Melbourne, Parkville, VIC 3010 Australia; 2 Department of Chemical Engineering, Monash University, Clayton, VIC 3800 Australia

**Keywords:** Chemical engineering, Heterogeneous catalysis, Carbon capture and storage

**replying to** F. de Meyer et al. *Nature Communications* 10.1038/s41467-023-39694-w (2023)

Since the signing of the Paris Climate Accords in 2015, the global effort for widespread deployment of carbon dioxide (CO_2_) capture facilities has accelerated. Despite the technical developments of conventional amine solvent technologies, only a limited number of these CO_2_ separation plants are operational at an industrial scale, aiding in the observed stagnation of annual CO_2_ emissions over the last decade. Such a lagging performance demonstrates the necessity to employ a diverse portfolio of new CO_2_ capture technologies and increase the number of large-scale capture facilities by at least an order of magnitude, to keep the temperature rise below 1.5–2.0 °C by 2050. Therefore, it is not reasonable to insist on the exclusive use of currently available industrial technology, as de Meyer has commented, for our recently published catalytic-solvent regeneration technology. The aim of the original publication is to develop a CO_2_ capture process that can be powered via renewable sources such as solar hot water, rather than steam which requires the use of fossil fuels.

The basis for de Meyer’s argument is that the desorption process is fundamentally controlled by equilibrium temperature, not by the kinetics of desorption. This statement is correct in relation to the currently applied amine circuit, in which the desorption process is typically controlled by equilibrium at ~120 °C, but not for a desorption process operating at 88 °C, which is the basis of our work.

de Meyer points out that the role of a high regeneration temperature in industrial CO_2_ desorption is twofold; (i) obtaining a lean solvent with an acceptably low CO_2_ loading and (ii) generating steam to strip CO_2_ and run a two-phase stripping column. Accordingly, both equilibrium and kinetics are favorable in industrial operations at ~120 °C. While this is correct, this type of operation is incompatible with renewable energies as excessive amounts of energy are consumed due to the latent heat of evaporation at the boiling point of the solution (~120–140 °C depending on the solution type and the operating conditions). Moreover, our recent findings reveal that many ‘green’ solvents such as amino acid solutions have a high degradation rate above 120 °C, hindering their deployment in emerging applications like direct air capture (DAC). We agree that the cyclic capacity will be smaller at a regeneration temperature of 88 °C compared to 120 °C, necessitating some larger equipment items. However, this difference is not catastrophic and may, in part, be compensated by adjusting the temperature of absorption and/or solution concentration. Most importantly, the reduced energy usage attained by avoiding solvent evaporation will outweigh the costs associated with larger equipment items, particularly if the energy required is provided by a solar source. Thus, de Meyer comparing the cyclic CO_2_ capacity of this technology to that of a currently available industrial system is not valid as it excludes solvent stability, type of energy required, its sustainability and life-cycle CO_2_ emissions.

**Fig. 1:**
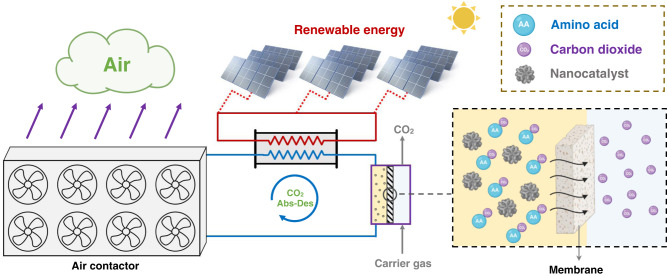
Schematic illustration of a solar-powered direct air capture integrated with water-dispersible nanocatalysts. The key is the low-temperature operation of the solvent membrane contactor for CO_2_ desorption. (Created with Biorender.com).

Although the issue around CO_2_ loading can be managed through engineering approaches, the slow kinetics of CO_2_ desorption at lower temperatures remains a serious challenge. To resolve this, two factors need to be focused on concurrently. The first is related to the reaction mechanism and the use of catalytic promoters to accelerate the kinetics of CO_2_ desorption at lower solvent regeneration temperatures, discussed in our recently published paper. The second is developing advanced liquid-gas separation techniques which are aligned with low-temperature solvent regeneration conditions. De Meyer has incorrectly assumed that our article is aimed at implementing CO_2_ capture using a traditional amine circuit with the same process conditions and equipment items, with just the addition of a nanocatalyst. Notably, he failed to consider that current industrial CO_2_ stripper columns only operate efficiently when both liquid and gas phases are present, necessitating constant solvent evaporation at high temperatures. In contrast, the maximum operating temperature during catalytic solvent regeneration (i.e., 88 °C) is far below the boiling point of the solution (i.e., ~120–140 °C) which makes the utilization of conventional CO_2_ stripping columns impractical. As the use of solar-heated water enables a move away from a steam reboiler and overhead condenser in the regenerator, there is an opportunity to investigate and utilize other equipment items with improved efficiencies and reduced costs. Gas-solvent membrane contactors, for example, are potential alternatives to conventional CO_2_ stripping columns as they mimic their operation at lower temperatures, but have lower energy usage. Optimal operation of a process with a regeneration temperature at 88 °C will require a new set of design conditions, equipment and process designs that have the potential to be competitive and cheaper than existing technologies. Fig. 1 presents a schematic of how water-dispersible nanocatalysts may be incorporated into a ‘next generation’ CO_2_ separation circuit. As indicated, different process conditions, equipment selections and perhaps CO_2_-containing gas streams will be used to fully exploit the potential of reduced desorption temperatures associated with the water-dispersible nanocatalysts developed in our paper^1^.

In conclusion, this article is focused on the future of solvent-based CO_2_ separation processes, rather than improving the current industrial CO_2_ capture process. Our idea is to develop a solar-powered CO_2_ capture unit without any dependency, direct or indirect, on fossil fuels. de Meyer’s assertion that using a solar-powered CO_2_ capture unit with water-dispersible nanocatalysts cannot lead to a green and low-cost unit is a simplification, and is mistakenly based on the assumption that water-dispersible nanocatalysts are added directly to a process operating in a conventional manner. de Meyer’s arguments only focus on the “Equilibrium CO_2_ Loading” and fail to consider opportunities provided by sustainable and renewable energy resources in the future of CO_2_ capture. It is clear that the current industrial technology is not viable for widespread and long-term installations, and the development of other technologies is crucial.
